# Role of Estrogen Receptor Signaling in Breast Cancer Metastasis

**DOI:** 10.1155/2012/654698

**Published:** 2011-12-19

**Authors:** Sudipa Saha Roy, Ratna K. Vadlamudi

**Affiliations:** Department of Obstetrics and Gynecology, The University of Texas Health Science Center at San Antonio, San Antonio, TX 78229, USA

## Abstract

Metastatic breast cancer is a life-threatening stage of cancer and is the leading cause of death in advanced breast cancer patients. Estrogen signaling and the estrogen receptor (ER) are implicated in breast cancer progression, and the majority of the human breast cancers start out as estrogen dependent. Accumulating evidence suggests that ER signaling is complex, involving coregulatory proteins and extranuclear actions. ER-coregualtory proteins are tightly regulated under normal conditions with miss expression primarily reported in cancer. Deregulation of ER coregualtors or ER extranuclear signaling has potential to promote metastasis in ER-positive breast cancer cells. This review summarizes the emerging role of ER signaling in promoting metastasis of breast cancer cells, discusses the molecular mechanisms by which ER signaling contributes to metastasis, and explores possible therapeutic targets to block ER-driven metastasis.

## 1. Introduction

The steroid hormone, estradiol, plays an important role in the progression of breast cancer, and a majority of the human breast cancers start out as estrogen dependent and express the estrogen receptor (ER). The biological effects of estrogen are mediated by its binding to one of the structurally and functionally distinct ERs (ER*α* and ER*β*) [1]. Endocrine therapy using Tamoxifen, a selective estrogen receptor modulator [2], and aromatase inhibitors, which ablate peripheral estrogen synthesis, has been shown to substantially improve disease-free survival [3]. Endocrine therapy has also been shown to have a positive effect on the treatment of ER-positive breast cancer [4]. Despite these positive effects, initial or acquired resistance to endocrine therapies frequently occurs with tumors recurring as metastatic. Tumor metastasis comprises a series of discrete biological processes that moves tumor cells from the primary neoplasm to a distant location [5] and involves a multi-step cascade of coordinated cell adhesion and contractility as well as proteolytic remodeling of the extracellular matrix (ECM) [6, 7]. Even though substantial information is available on the process of metastasis, the molecular basis of breast cancer progression to metastasis and the role of ER*α* signaling in this process remain poorly understood. A few early studies suggested a negative effect of ER*α* signaling on motility and invasion of cells [8, 9], while several recent studies showed a positive effect of ER signaling on motility [10–14]. In this review, we summarized the emerging evidence for the role of ER*α* signaling in breast cancer progression to metastasis and discuss the possibility of targeting ER*α* signaling crosstalk with cytosolic kinases as a possible additional therapeutic target for treating/preventing ER-positive metastatic breast cancer.

## 2. ER***α*** Signaling Mechanisms

ER*α* is the major ER subtype in the mammary epithelium and plays a critical role in mammary gland biology as well as in breast cancer progression [15, 16]. The ER*α* comprises an N-terminal AF1 domain, a DNA-binding domain, and a C-terminal ligand-binding region that contains an AF2 domain [17]. Upon the binding of estrogen to ER*α*, the ligand-activated ER*α* translocates to the nucleus, binds to the responsive element in the target gene promoter, and stimulates gene transcription (genomic/nuclear signaling) [18, 19]. Emerging evidence suggests that ER signaling is complex, involving coregulatory proteins and also genomic actions and extranuclear actions [20, 21].

 Multiprotein complexes containing coregulators assemble in response to hormone binding and activate ER-mediated transcription [18]. The ER*α* transcriptional outcome is regulated by dynamic chromatin modifications of the histone tails, and the ligand-bound ER*α* facilitates these modifications via coregulator recruitment [22]. For example, coactivators like SRC-1, amplified in breast cancer (AIB1), and CBP have been shown to possess histone acetyltransferase activity, whereas corepressors, such as NCOR and MTA1, are associated with histone deacetylases [20, 23]. It is generally accepted that some of the diverse functions of E2 depend on differential recruitment of coregulators to the E2-ER complex [24]. Even though coregulators modulate ER functions, each coregulator protein appears to play an important but not overlapping function *in vivo* [25–27].

 Emerging findings suggest that ER-coregulatory proteins have potential to be differentially expressed in malignant tumors and that their functions may be altered, leading to tumor progression [28]. *In vivo* studies using wild type (WT) and SRC3/AIB1^−/−^ mice harboring the mouse mammary tumor virus-polyomavirus middle T (PyMT) transgene (Tg) revealed that AIB1 knock down significantly reduces lung metastasis but not mammary tumorigenesis. Compared with *WT*/*PyMT* mice, Tg *SRC*-1^−/−^/*PyMT* mice had intravasation of mammary tumor cells. In addition, the frequency and extent of lung metastasis were drastically lower in the Tg mice than in the WT mice [29]. Another study using Tg *SRC*-1^−/−^ mice reported that deficiency of SRC-1 coregulator increases MMTV-neu-mediated tumor latency and differentiation-specific gene expression and decreases metastasis [30]. Collectively, these emerging findings implicate the role of the ER*α*-coregulator-associated activities/functions in breast cancer metastasis.

## 3. ER***α*** Genomic Actions and Metastasis

Within the last decade, research has provided substantial data to suggest that alteration in cellular concentration or genetic dysfunction of coregulators can contribute to a pathologic outcome by modulating ER genomic actions and has potential to drive cancer cell proliferation and metastasis [31]. Loss of the epithelial adhesion molecule E-cadherin is implicated with a critical role in metastasis by disrupting intercellular contacts, an early step in metastatic dissemination [32]. Functional or transcriptional loss is commonly associated with an invasive and poorly differentiated phenotype [33]. Deregulation of ER-coregulator signaling can lead to aberrant expression of Snail, resulting in the loss of expression of E-cadherin and invasive growth. For example, MTA1, a commonly deregulated coregulator in breast cancer, promotes transcriptional repression of ER, leading to metastatic progression [34]. The ER*α* coregulator (AIB1) amplified in breast cancer has been shown to promote breast cancer metastasis by activation of PEA3-mediated matrix metalloproteinase 2 (MMP2) and MMP9 expression [35]. SRC-1, another ER coregulator, has also been shown to promote breast cancer invasiveness and metastasis by coactivating PEA3-mediated Twist expression [36]. Recent studies have found deregulation of the ER coregulator PELP1 in invasive and metastatic breast tumors [37, 38]. Recent studies using PELP1 overexpression and knockdown demonstrated that PELP1 plays an important role in ER*α*-positive metastasis [10]. Collectively, these studies indicate that ER*α* and ER coregulators modulate expression of genes involved in metastasis.

## 4. ER***α*** Extranuclear Actions and Metastasis

**Figure 1:**
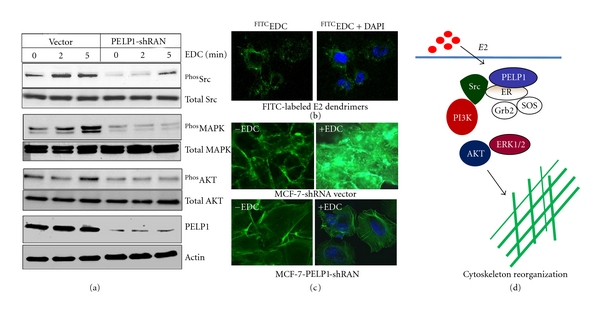
ER-extranuclear signaling promotes actin reorganization via ER coregulator PELP1. (a) MCF7 shRNA vector control and MCF7-PELP1-shRNA cells were cultured in 5% DCC serum containing medium treated with or without estrogen dendrimers (EDC). The activation of signaling pathways was analyzed by Western blotting of total protein lysates with phospho-specific antibodies. (b) MCF7 cells were treated with FITC-labeled EDC and localization of EDC was analyzed by confocal microscopy*. Green; EDC; Blue, DAPI.* (c) MCF7 or MCF7-PELP1-shRNA cells were treated either with E2 or EDC and the F-actin status was analyzed by phalloidin staining and visualized by confocal microscopy. (d) Schematic representation of estrogen-mediated extranuclear signaling. Adapted from [10].

Emerging evidence suggests that the ER*α* participates in extranuclear signaling [39]. ER*α* activation, by E2, induces key features of motile cells including rapid cytoskeletal reorganization and the development of specialized structures including fillopodia and ruffles [37]. To establish the role of E2-mediated extranuclear actions, researchers developed E2-Dendrimers (EDCs), which are nanoparticles coated with estrogen. These EDCs uniquely localize in the membrane and cytoplasm, preferably activating ER*α* extranuclear signaling. Using these EDCs, researchers have demonstrated that ER*α* extranuclear pathways have distinct biological outcomes [40]. Our laboratory using EDCs provided further evidence that ER*α* extranuclear signaling has the potential to contribute to the breast cancer cell motility (Figure 1) [10]. ER*α* extranuclear signaling promotes stimulation of the Src kinase, mitogen-activated protein kinase (MAPK), phosphatidylinositol 3-kinase (PI3K), and protein kinase C pathways in the cytosol (10, 11). Recent studies identified PELP1 as one of the components of the ER*α* signalosome in the cytoplasm, and estrogen-mediated extranuclear signaling promotes cytoskeleton reorganization via ER-Src-PELP1-PI3K-ILK1 pathway [10]. Many of the kinases activated by ER*α* extra-nuclear signaling are implicated in breast cancer metastasis. For example, ERK and protein kinase B (AKT) phosphorylation play important roles in breast cancer cell migration [14], and Src and ILK1 kinases play critical roles in invasion and metastasis of breast cancer cells [41, 42].

In addition to ER*α* interactions with cytosolic kinases, few other mechanisms by which the ER*α* activates extranuclear signaling have been reported. Membrane-bound ER*α* has been reported to be associated with growth factor receptors such as IGF-1R, EGFR, and HER2; such interactions play a role in cytoskeleton reorganization [43]. Dysregulation of HER2 in breast cancer cells enhances the expression of an isoform of MTA1 (MTA1s), which promotes the cytoplasmic sequestration of ER*α* leading to constitutive activation of MAPK. These study findings implicate the regulation of the cellular localization of ER*α* by MTA1s as a mechanism for enhancing ER*α* extranuclear actions by nuclear exclusion [44]. Recent studies also found that the ER*α* was methylated via posttranslational modifications, and methylated ER*α* was predominantly present in the cytoplasm, suggesting that deregulation of arginine methylases may have consequences in activation of ER*α* extranuclear actions [45]. Collectively, these emerging results suggest that ER extranuclear signaling has the potential to promote breast cancer cell migration and metastasis.

## 5. ER***α*** Regulation of Metastasis

Metastases spawned by malignant tumors that have acquired increased invasiveness are responsible for almost all breast-cancer-related morbidity and mortality. The majority of ER*α*-positive cells retain their ER*α* and respond positively to initial endocrine therapy for the treatment of advanced metastatic disease. Several recent studies have detected the presence of ER*α* expression in metastatic tumors [46–48]. A correlation between ER*α*-positive tumors and the development of bone metastasis has been observed clinically [49, 50]. Many metastatic tumors retain ER*α*. If primary tumors are ER*α* positive, greater than 80% of the lymph node metastases, and 65–70% of distant metastases retain ER*α* [46, 47]. A clinical correlation has also been reported between ER*α*-positive tumors and the development of bone metastasis [49, 50]. ER*α* signaling has also been shown to enhance lung metastasis [51]. In addition, ER*α*-mediated signaling has enhanced lung metastasis by promoting host-compartment response [51]. These emerging findings suggest that ER*α* signaling plays a role in metastasis.

## 6. ER***β*** Regulation of Cell Migration and Metastasis

ER*β*, similar to ER*α*, also functions as a transcription factor that mediates different physiological responses to estrogen signaling. However, the physiological consequences of ER*β*-mediated transcriptional regulation are distinct from those of ER*α* [1]. A number of recent studies suggest that an increase in ER*β* expression decreases cell proliferation and that ER*β* has antiproliferative (tumor suppressor) functions [52–54]. Reduced expression of ER*β* was reported in invasive breast cancer [55], and ER*β* expression is associated with less invasive and proliferating tumors [56]. Downregulation of ER*β* is shown to promote epithelial-to-mesenchymal transition (EMT) in prostate cancer cells [57]. A recent study using breast cancer model cells provided evidence that ER*β* expression was associated with less cell migration. Mechanistic studies indicated that ER*β* affects integrin expression and clustering and consequently modulates adhesion and migration of breast cancer cells [58]. Collectively, the emerging evidence in various model cells (including ovary and prostrate) suggests that ER*β* signaling may promote antimigratory and anti-invasive responses; however, future studies using breast models are needed to further validate these findings.

## 7. Estrogen Regulation of EMT

EMT constitutes the loss of hallmark structures and physiologic properties associated with the epithelia and the gain of new properties, including migratory and invasive growth patterns [59]. Loss of E-cadherin is a key initial step in the transdifferentiation of epithelial cells to a mesenchymal phenotype, which occurs when tumor epithelial cells invade the surrounding tissues [60]. Evolving evidence suggests that estrogen signaling can influence EMT and ER*α* signaling crosstalk with several EMT regulators such as Snail and Slug. ER*α* directly binds to and regulates the promoter of metastasis tumor antigen (MTA) 3 that suppresses *Snail*, a gene implicated in EMT transition [61]. ER*α* downregulates *Slug* transcription by the formation of a corepressor complex involving HDAC1 (histone deacetylase 1) and N-CoR (nuclear receptor co-repressor) [62]. Estrogen promotes down-regulation of E-cadherin via transcriptional regulation by recruitment of corepressors such as scaffold attachment factor B [63]. Estrogen plays an important role in cytoskeletal rearrangements mediated by delocalization of E-cadherin [64]. Furthermore, a recent study found that E2 promotes reversible EMT-like transition as well as collective motility in ER*α*-positive cells [65]. Estrogen-regulated EMT is complex and is dependent on temporal expression patterns of MTA family members, cell-adhesion-essential regulators, and ER coregulators [66]. ER*α* signaling negatively regulates EMT by modulating MTA3 expression and thus promotes differentiation [61]. Collectively, these findings implicate that estrogen-mediated EMT depends on the cellular repertoire of ER*α* coregulators and EMT regulators and that their cross talk has potential to differentially affect breast cancer progression, leading to metastasis via EMT changes.

## 8. Tumor Microenvironment Regulation of ER Signaling

The metastasis signaling cascade is orchestrated through the activation of biochemical pathways that involve the tumor microenvironment. Stromal cells (fibroblasts, inflammatory cells, and endovascular cells) play important roles to create a supportive environment for tumor cell growth [67, 68]. Chemokines produced by stromal cells have potential to influence ER*α*-positive breast cancer progression to metastasis. The chemokine CXCL12/SDF-1 and its G-protein-coupled receptor CXCR4-mediated signaling pathways play important roles in the migration and invasion of breast cancer cells. Some evidence suggests that HER2-mediated breast tumor metastasis may involve HER2 and CXCR4 signaling pathway cross talk [69]. CXCR4 overexpression correlated with worse prognosis in patients and constitutive activation of CXCR4 in poorly metastatic ER-positive MCF7 cells led to enhanced tumor growth and metastasis. The results from this study showed that enhanced CXCR4 signaling is sufficient to drive ER*α*-positive breast cancers to a metastatic and endocrine-therapy-resistant phenotype via increases in MAPK signaling [70].

 The intratumoral levels of estrogens and growth factors are regulated by the tumor-stromal interactions in the tumor microenvironment [71]. Cross talk between the tumor and stromal cells promote expression of aromatase, a key enzyme in E2 biosynthesis, resulting in intra-tumoral estrogen production in postmenopausal breast tumors [72]. Tumor-stromal cross talk regulates aromatase gene expression via the production of various factors such as COX2, tumor necrosis factor-*α*, interleukin-6, and interleukin-11 [71]. Tumor-stromal interactions also contribute to the expression of growth factors such as EGF and IGF-1, which activate the ER*α* through growth factor receptor cross talk, leading to ER*α*-positive breast cancer progression [73].

## 9. ER Signaling Components as Potential Biomarkers for Predicting Metastasis

ER*α* status is routinely used in the clinic for treatment selection; however, additional markers are urgently needed to predict metastasis. Considering the evolving significance of ER*α* coregulators (SRC family members such as SRC-3/AIB1) in mammary tumor invasion and metastasis [74], SRC-3 status could be used as a diagnostic biomarker. Similarly, expression of the ER coregulator PELP1 is deregulated in metastatic breast tumors [37], and PELP1 protein expression is an independent prognostic predictor of breast cancer-specific survival and disease-free survival [38]. Since PELP1 plays a critical role in estrogen-mediated extranuclear signaling, these findings suggest that PELP1 could be used as a potential biomarker for predicting ER-driven metastasis. Several studies using various Src kinase inhibitors and dominant-negative mutants demonstrated that inhibiting c-Src activity decreased the metastatic potential of breast cancer cells [75]. Given the role of Src kinase in ER signaling, phosphor-c-Src is an attractive biomarker for predicting breast cancer metastasis in conjunction with other prognostic factors. Few recent preclinical studies using Src inhibitors confirmed the downstream target of Phos-Src and -FAK and could be possible diagnostic markers [76]. Because AKT signaling is implicated in invasive ductal carcinoma of the breast and implicated in ER*α*-mediated extranuclear actions leading migration/invasion, Phospho AKT (pAKT) status could be a potential biomarker in the prediction of therapeutic response in invasive ductal carcinoma of the breast [74]. Even though these emerging findings suggest ER*α*-signaling molecules as potential biomarkers, additional studies using a large set of human tumor samples are needed to clearly establish them as prognostic markers.

## 10. Therapeutic Targeting of ER***α*** Signaling for Blocking Metastasis

The emerging significance of the ER*α* in the metastatic cascade indicates novel possibilities for therapeutic targeting of specific ER*α* signaling components that mediate migration, invasion, and EMT. A large portion of metastases retain their ER*α* when the primary tumors are ER*α* positive. Several recent studies detected the presence of ER*α* and aromatase expression in metastatic tumors [46–48]. We envision that the therapies targeting ER signaling axis leading to metastasis are more suitable for early stage patients who have tumors that are amenable to biopsy and IHC analysis. Potential markers of ER*α* signaling that are implicated in metastasis (including kinases such as Src, AKT, and PI3K and coregulators such as PELP1, AIB1, and SRC-1) could be used in addition to traditional ER*α* status to identify this subset of patients.

 Aromatase is recognized as a potent target in endocrine therapy for the treatment of postmenopausal breast cancers [73]. Because some metastases retain their ER*α* signaling, screening of patients with advanced breast cancer for expression of ER*α*, ER-coregulators, and aromatase may provide a rationale for the development of customized treatment of a subset of patients with ER*α*-positive and aromatase-positive cancer. These patients could be treated with an aromatase inhibitor (Letrozole) that ablates peripheral estrogen synthesis and ER*α* degraders/signaling blockers for their ER*α*-positive metastatic tumors.

 Because ER*α* and ER*β* have different physiological functions and have ligand-binding properties that differ enough to be selective in their ligand binding, opportunities now exist for testing of novel ER subtype-specific, selective ER modulators [77]. Several synthetic or novel natural compounds derived from plant materials have the potential to function as ER*β* agonists [54, 78], and these compounds may have utility in augmenting ER*β* tumor suppressive functions.

If ER*β* can hamper the regulation of ER*α* and inhibit the proliferation as well as affect the crosstalk with growth factors and their receptors, testing of ER*β* agonist in combination with other endocrine therapies will provide a novel means to target ER*α*-driven metastasis. Recent studies found a therapeutic efficacy using ER*β* agonists in combination with aromatase inhibitors, and this strategy may be useful in treating aromatase-inhibitor-(AI-) resistant metastatic breast cancer [79].

 ER*α*-positive metastasis has been associated with chemokine signaling through SDF-1-CXCR4. Therefore, CXCR4 signaling is a rational therapeutic target for the treatment of ER-positive advanced breast carcinomas [70]. Integrin-linked kinase (ILK) is a nodal molecule in many molecular pathways that are implicated in cancer metastasis. Recent evidence suggests that ER extranuclear signaling utilizes the ILK axis [10]; therefore, ILK inhibitors such as QLT-0267 could be used to curb motility of breast cancer cells [80]. Since arginine methylation is implicated in ER*α* extranuclear signaling, blocking arginine methylases could be a possible therapeutic target. Compounds such as guanidine-nitrogen-substituted peptides or the thioglycolic amide RM65 may be useful to block this pathway [81, 82]. SRC3/AIB1 is frequently amplified or overexpressed in human breast cancer and is implicated in breast cancer progression to advanced ER*α*-positive tumors. Mechanistic studies showed AIB1 overexpression activates the mammalian target of rapamycin (mTOR), and activation of mTOR pathway is critical for AIB1-driven tumorigenesis [83]. Recent studies suggest that mTOR inhibition and ER-targeted endocrine therapy may improve the outcome of the subset of patients with ER-positive breast cancers overexpressing AIB1 [84].

 Emerging evidence suggest that Src participates in ER*α* extranuclear actions and its wide deregulation in breast tumors suggests that it could be a potential candidate for treating ER*α*-positive metastasis [85]. The fact that Src can mediate interactions between the ER*α* and growth-factor-signaling pathways is of particular importance because cross talk between these pathways is implicated in activation of ER*α* extranuclear signaling leading to cell migration and invasion [10]. Further, the ability of the Src axis to promote local estrogen synthesis via aromatase activation has potential to form an autocrine loop of ER*α* signaling leading to tumor cell proliferation and metastasis [86]. Thus, blocking the Src axis could block ER*α* signaling at multiple fronts and thus reducing the ability of the ER*α* to promote metastasis. Recent studies found that inhibition of the Src family tyrosine kinases using inhibitors such as dasatinib can block ER*α*-mediated extranuclear actions leading to cell migration and invasion [10]. Therefore, it is tempting to speculate that combination of hormonal therapy with dasatinib, an orally available inhibitor of Src family tyrosine kinases that is currently approved for clinical trials to treat solid tumors [87–89], may be useful in curbing breast cancer metastases.

## 11. Conclusions/Significance

**Figure 2:**
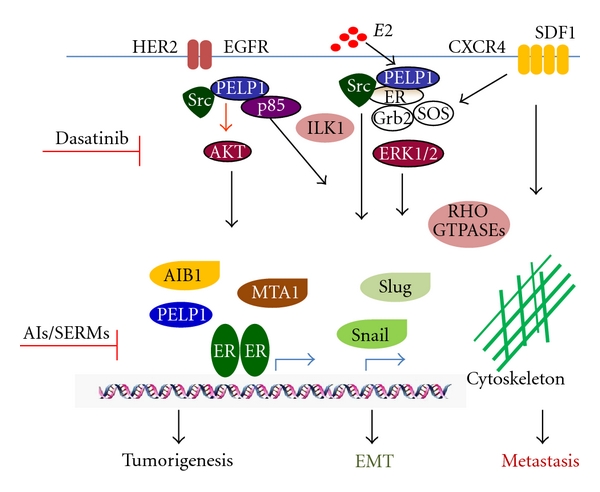
Schematic representation of hormonal regulation of metastasis. ER*α*-mediated signaling involves nuclear as well as extranuclear actions and growth factor signaling cross talk. Estrogen signaling has the potential to activate extranuclear signaling that activates several kinase cascades, which have potential to alter cytoskeleton, EMT and enhance cell migration. Deregulation of ER*α*-mediated signaling crosstalk will have implications in estrogen-mediated tumor progression to metastasis.

The most deadly aspect of breast cancer is its ability to spread or metastasize. Recent mechanistic studies have increased our understanding and highlight a role of estrogen-induced rapid ER*α* extranuclear signaling in facilitating the metastatic process. This signaling pathway thus provides new targets for therapeutic intervention. During progression from tumorigenesis to invasion, tumor cells trigger signals that activate ER*α*-extranuclear-signaling pathways, leading to enhanced cell migratory functions and metastasis, thus ER extranuclear signaling represents an important target for metastatic control of ER*α*-positive tumors (Figure 2). Since multiple signaling pathways in addition to estrogen are involved in activating ERs, combination therapies using both endocrine and nonendocrine agents that block different pathways may have better therapeutic effects and may delay the development of estrogen-driven metastasis. Future studies identifying the molecular mechanisms of ER*α* signaling contributing to ER*α*-driven metastasis as well as examining the prognostic/diagnostic significance of ER*α* signaling components using a larger sample size of tumors is warranted. Further, elucidation of the pathologic roles of ER*α* extranuclear signaling in metastasis will have important implications for development of novel breast cancer therapeutics and in the development of the next generation of selective ER modulators.
